# The vascular and neurogenic factors associated with erectile dysfunction in patients after pelvic fractures

**DOI:** 10.1590/S1677-5538.IBJU.2014.0170

**Published:** 2015

**Authors:** Yong Guan, Sun Wendong, Shengtian Zhao, Tongyan Liu, Yuqiang Liu, Xiulin Zhang, Mingzhen Yuan

**Affiliations:** 1 ShanDong University, Jinan, China; 2 Department of Urology, the Second Hospital of ShanDong University, Jinan, China; 3 The Second Hospital of ShanDong University, Jinan, China

**Keywords:** Erectile Dysfunction, Urethra, Pelvis, Penis

## Abstract

Erectile dysfunction (ED) is a common complication of pelvic fractures. To identify the vascular and neurogenic factors associated with ED, 120 patients admitted with ED after traumatic pelvic fracture between January 2009 and June 2013 were enrolled in this study. All patients answered the International Index of Erectile Function (IIEF-5) questionnaire. Nocturnal penile tumescence (NPT) testing confirmed the occurrence of ED in 96 (80%) patients on whom penile duplex ultrasound and neurophysiological testing were further performed. Of these ED patients 29 (30%) were demonstrated only with vascular abnormality, 41 (42.7%) were detected only with neural abnormality, 26 (27.1%) revealed mixed abnormalities. Of the 55 patients (29+26) with vascular problems, 7 patients (12.7%) with abnormal arterial response to intracavernous injection of Bimix (15mg papaverine and 1mg phentolamine), 31 (56.4%) with corporal veno-occlusive dysfunction and 17 (30.9%) had both problems. Of the 67 (41+26) patients with abnormal neurophysiological outcomes, 51 (76.1%) with abnormal bulbocavernosus reflex (BCR), 20 (29.9%) with pathological pudendal nerve evoked potentials (PDEPs) and 25 (37.3%) with abnormal posterior tibial somatosensory nerve evoked potentials (PTSSEPs). Our observation indicated that neurogenic factors are important for the generation of ED in patients with pelvic fracture; venous impotence is more common than arteriogenic ED.

## INTRODUCTION

Erectile dysfunction (ED) is defined as the inability to achieve or maintain an erection adequate for sexual satisfaction (1). It has been reported that 3% of cases of ED may result from pelvic fractures or perineal blunt trauma (2). The incidence of ED ranges from 20% to 84% in patients with urethral injury secondary to perineal trauma or pelvic fractures (3). ED caused by pelvic fractures, especially associated with urethral injuries, is more common than previously described (2). It is assumed that ED caused by such reasons is due to lesions of the cavernous nerves or branches of the internal pudendal arteries that pass in close proximity to the pelvic bones and posterior urethra. The intimate relationship of the soft tissues and the bony pelvic ring result in a high risk of concomitant local injury associated with fractures of the pelvis (4). Even without severe urological injury, damage to the delicate vascular and nervous tissues supplying the genitalia can result in sexual dysfunction (3, 5). While knowledge from pelvic microscopic anatomy and erectile physiology provided insights for alternative pathways in the development of ED (2, 6, 7), few studies have been designed to analysis the exact pathophysiological factors in this type of ED patients (8, 9), particularly no study was performed to differentiate the neurogenic ED from vasculogenic ED and it was just assumed that patients with normal vascular response are neurogenic ED (3, 10); furthermore, the number of the patients observed in most of the reports are not big enough which for some extent may impact the interpretation of the results (3, 11). Therefore, the main purpose of our study was to evaluate the vascular and neurogenic factors associated with ED in a relatively large population of patients after pelvic fracture.

## MATERIALS AND METHODS

### General information of the patients

120 patients who were admitted to the Second Hospital of Shandong University between January 2009 and June 2013 for the complaint of ED were enrolled in this study. All patients had a history of pelvic fractures associated with urethral injuries and were submitted to urethral realignment by traction. According to patient history, they were free from ED before the injury. The age ranged from 21 to 48 years old (mean age 37.6±6.3). Imaging studies taken at admission (e.g., pelvic radiographs, computed tomography scans) were used to classify the injury according to the modified Tile's classification and Denis’ classification for sacral fractures (12–14). Based on the criteria we classified these patients into type A (Stable, minimally displaced), type B (rotationally unstable, vertically stable) and type C (rotationally and vertically unstable).

### Blood tests

To exclude hormonal factors related with ED the levels of testosterone (T), estradiol (E2), luteinizing hormone (LH) and follicle-stimulating hormone (FSH) in the peripheral blood plasma were measured by radioimmunoassay. Blood lipid, blood glucose level and blood pressure were also checked after admission.

### IIEF-5 questionnaires

IIEF-5 questionnaires were answered by all patients. A modification of the method developed by Cappelleri was used for grading of ED into four categories: no ED (scores 26-30), mild ED (scores 17-25), moderate ED (scores 11-16) and severe ED (scores 6-10) (15).

### Nocturnal penile tumescence (NPT) test

NPT tests were performed using the RigiScan® device in all the patients. To ensure a restful night sleep the patient was asked to avoid napping, caffeine or alcohol intake and to evacuate the bladder prior to going to sleep. The data were collected each morning. The test was conducted over two consecutive nights in order to avoid the “first night effect”. Normal nocturnal erectile function was defined as at least 3 tumescence periods lasting more than10 minutes with rigidity at the penile tip of at least 70% (3).

### Duplex ultrasonography and cavernosography

**Figure 1:**
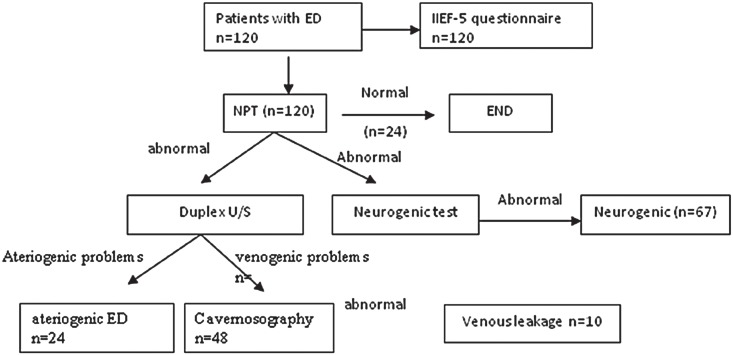
Flow diagram for evaluation of ED patients. All patients answered IIEf-5 questionnaire and were submitted to npT. Duplex ultrasonography and neurophysiological tests were performed if problems were detected with npT. cavernosography was undertaken to determine if there was venous leakage in patients suspected with venogenic ED.

Patients with abnormal NPT outcome were submitted to duplex ultrasonography test (GE LOGIQ9, America) (Figure-1). Patients received a single intracavernous injection of Bimix (15mg papaverine and 1 mg phentolamine). The erectile response was evaluated for tumescence and rigidity by palpation of the penis. The erectile status after intracavernous injection was also compared with that demonstrated at home before trauma in order to obtain the best quality erection (16). The penis was scanned by a ventral approach at the base with the probe held transversally or in an oblique-longitudinal position. Peak systolic velocity (PSV), end diastolic velocity (EDV) and resistance index (RI) within the cavernosal arteries were measured. Patients with PSV greater than 35cm/sec were considered with a normal arterial response, while less than 25cm/sec signified severe arterial insufficiency. Corporal veno-occlusive dysfunction was defined as EDV >5cm/sec and RI <0.85 (3, 17, 18). If venogenic ED was highly suspected, cavernosography was performed by intra-cavernous injection of contrast following administration of vasodilators (15mg papaverine and 1 mg phentolamine).

### Neurophysiological tests

Posterior tibial somatosensory nerve evoked potentials (PTSSEPs), pudendal nerve evoked potentials (PDEPs) and the bulbocavernosus reflex (BCR) test were conducted for all the ED patients confirmed by NPT test. PTSSEPs and PDEPs were performed according to the International Federation of Clinical Neurophysiology (IFCN) standards (19). The latency of cortical P40>45ms or left–right difference >2.5ms were considered abnormal in PTSSEPs test. P40 latency>44.1 ms was considered pathological in PDEPs test (20). BCR test was performed by applying electrical pulses on the penis and the responses were recorded from both bulbocavernosus muscles with concentric needle electrodes (20, 21). Abnormal results include absent responses, response latency >37ms and interside differences >1.5ms.

## RESULTS

All of the patients had normal levels of blood hormones including testosterone, estradiol, LH and FSH. Blood lipid, blood glucose and blood pressure level of these patients were also normal. According to the modified Tile's classification and Denis’ classification for sacral fractures, 72 patients (60.8% of 120) were classified as type A fracture, 35 patients (29%) were type B, and 12 patients (10%) were type C (Table-1).

**Table 1:** Patients distribution based on the type of pelvic fracture.

	Type A	Type B	Type C
Number of patients.	73	35	12
%	60.8% (73 of 120)	29.2% (35 of 120)	10.0% (12 of 120)

All of the patients answered the IIEF-5 questionnaire and 115 (95.8%) were considered with ED based on the Cappelleri's grading criteria (15); of them 16 patients were mild ED, 34 patients were mild to moderate ED, 47 patients were moderated ED and 18 patients had severe ED (Table-2).

**Table 2:** Grading and distribution of ED patients based on IIEf-5 scores.

ED Status	Theoretical EF Domain Scores*	The Number of Patients (%)
Without ED	(26-30)	5 (4.2%)
With ED	(<25)	115 (95.8%)
Mild ED	(22-25)	16 (13.3%)
Mild to moderate ED	(17-21)	34 (28.3%)
Moderate ED	(11-16)	47 (39.2%)
Severe ED	(6-10)	18 (15.0%)

* Criteria from Cappelleri et al. ^13^.

**Figure 2:**
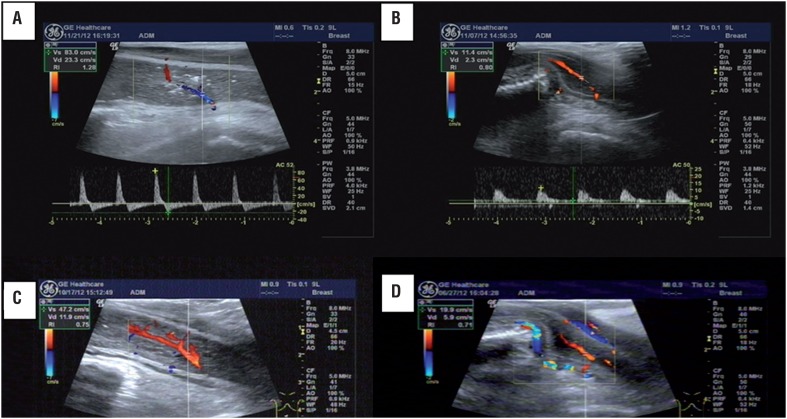
Abnormal arterial or venous responses to intracavernous injection of Bimix in ED patients. patients received a single intracavernous injection of Bimix (15 mg papaverine and 1 mg phentolamine) then ultrasonography was performed. (a) peak systolic velocity (psv)>35cm/sec and the resistance index (RI)=1 in a patient indicating a normal arterial and venous response. (b) In one ED patient psv<35cm/sec with the end-diastolic velocities (EDv)<5cm/sec suggesting abnormal arterial response. (c) in another ED patient psv>35cm/sec with EDv>5cm/sec indicating veno-occlusive dysfunction. (d) psv<35cm/sec with EDv>5cm/sec suggesting both arterial and venous abnormal responses.

**Figure 3:**
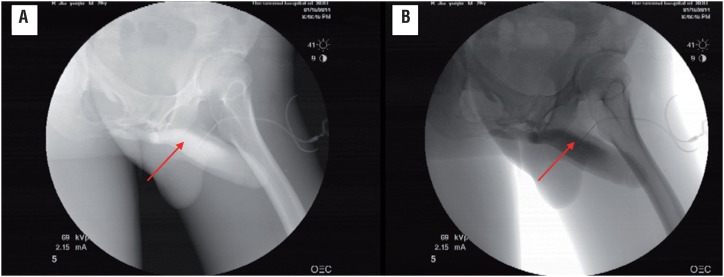
Venous leakage revealed by cavernosography in venogenic ED patients. contrast was injected intracavernously following administration of 15 mg papaverine and 1 mg phentolamine. solid arrow indicates leakage from the vein. (a) the positive film (b) the negative film.

NPT tests showed normal nocturnal erections in 24 (20.0%) patients, abnormal nocturnal erections in 96 patients (80.0%) in whom further Duplex ultrasonography, cavernosography and neurophysiologic testing revealed organic lesions (Figure 1 and Table-3). Penile duplex Doppler ultrasonography detected abnormal responses to intracavernous injection of Bimix in 55 patients (45.8% of 120) (Table-3), of them 29 patients only with abnormal vascular response, 26 with abnormal vascular as well as neurogenic problems, 7 patients (12.7%) with abnormal arterial response, 31 (56.4%) with corporal veno-occlusive dysfunction and 17 (30.9%) had both problems (Table-4, Figure-2). Of the 31 patients with corporal veno-occlusive dysfunction, there were 5 patients with penile venous leakage revealed via cavernosography (Figure-3). Abnormal neurophysiologic outcomes were seen in 67 patients, of them 41 patients were only with abnormal neurophysiological outcome and 26 with both abnormal neurophysiologic outcome and vascular problem. Pathological PDEP responses were seen in 20 patients (29.9% of 67), abnormal latencies of PTSSEPs were observed in 25 patients (37.3%), problematic BCR were found in 51 patients (76.1%) (Table-5); of them 9 patients (17.6%) with bilaterally abnormal, 37 (72.5%) with unilaterally abnormal and 5 (9.8%) with no response (Table-6).

**Table 3:** Distribution of ED patients based on etiology.

Origin	NPT	Vasculogenic ED			Neurogenic ED	Vasculogenic and Neurogenic
		Arteriogenic	Venogenic	Mixed		
Number	96	3	16	10	41	26

One of the neurophysiologic tests (BCR, PDEP or PTSSEPs) was abnormal indicating the patients had neurogenic ED.

**Table 4:** Vasculogenic ED patient distribution.

	Arteriogenic	Venogenic	Mixed
Number of patient.	7	31	17
%	12.7% (7 of 55)	56.4% (31of 55)	30.9% (17 of 55)

These 55 vasculogenic ED patients include 29 only with vasculogenic and 26 with both neurogenic and vasculogenic origin.

**Table 5:** Neurogenic ED patient distribution.

	BCR	PDEP	PTSSEPs
Number of patients.	51	20	25
%	76.1% (51 of 67)	29.9% (20 of 67)	37.3% (25 of 67)

These 67 patients included 41 only with neurogenic and 26 with both neurogenic and vasculogenic origin. One patient may have two or three abnormal outcomes.

**Table 6:** ED patients with abnormal BCR outcomes.

	Bilateral	Unilateral	No response
Number of patients.	9	37	5
%	17.6% (9 of 51)	72.5% (37 of 51)	9.8% (5 of 51)

## DISCUSSION

Pelvic fractures, particularly those associated with posterior urethral injury often cause ED. It had been reported ED occurred in 20-84% of cases when pelvic fractures were associated with urethral injury (3, 6, 10). In our study all of the patients had a history of pelvic fractures associated with urethral injuries; NPT test demonstrated 80% of those patients had organic ED. The criterion used in our study for NTP testing was the same as Shenfeld's report (3) and the incidence of ED (80%) was closer to 72% reported by them and was also similar to 84% reported by Flynn et al. (22). Our observation that neurogenic ED accounted for most of the ED patients is consistent with other reports indicating neurogenic factor is the principal etiology of organic ED associated with urethral injury (3, 10).

The IIEF was used as a self-administered questionnaire and proved an adequate tool in bringing forward the latent expectations of the patients; it might be used at the time of rehabilitation to identify those patients who would need further evaluation and treatment (23). The limitation of self-administered questionnaires is that they do not distinguish an etiologic basis for ED. In our study IIEF scores were used to assess the severity of ED and 95.8% of patients were considered with ED, compared with the 80% ED patients detected with NPT test indicating some of the ED patients might had a psychogenic origin.

Erections are initiated by a combination of psychic and physical stimuli, and erectile function is controlled by parasympathetic fibers originating from S2 to S4. These fibers travel through the pelvic nerve and the pelvic plexus to the cavernous nerve, which enters the corpora cavernosa. As these fibers pass through the pelvis, the nerves run in close proximity to the prostate and rectum, which makes them prone to injury during surgical procedures as well as after pelvic trauma. It has been speculated that the etiology of ED after pelvic fracture is neurovascular injury. Most previous studies that address the etiology of ED with pelvic fracture did not differentiate neurogenic from the vascular factors (3, 10, 24), they just indirectly concluded that most of the ED patients were neurogenic, since most of the ED patients had a normal arterial response to intracavernous injection of either Trimix (3) or Bimix (10). To our knowledge our study is the first report that electrophysiological testing was performed to identify the neurological pathologies related with ED after pelvic fracture. We found that 69.7% of ED patients had abnormal electrophysiological outcomes and were diagnosed as neurogenic ED. As mentioned above, penile erection is primarily an autonomic nerve function (cavernous nerve), but currently there are no sensitive and direct neurophysiologic tools to test on it. Electrophysiological tests including BCR, PTSSEPs and PDEPs were used to diagnose neurogenic ED in our study based on the following considerations: (1) while these tests mainly evaluate somatic nerve functions, there is increasing evidence in literature that the autonomic and somatic functions are anatomically and physiologically connected in the pudendal nerve (25, 26), therefore, abnormal outcomes of these tests particularly BCR and PDEPs can indirectly reflect the insufficiency of cavernous nerve mediated erection; (2) they are widely used for assessing neurological alterations related with ED in literature reports (20, 21, 27), for example, neurophysiological testing including BCR, PTSSEPs, PDEPs etc was used to detect peripheral neuropathy in ED patients (20). BCR was performed to predict the response of ED patients following radical prostatectomy to sildenafil citrate (28), BCR testing was used to detect neurogenic impotence in patients after radical prostatectomy (3, 29) no other neurological pathologies that are closely related with ED such as diabetic neuropathy, hypertension, hypercholesterolemia etc were detected in all the patients enrolled in our study, therefore, abnormal neurophysiologic findings must be pelvic fracture related. The detected rate of abnormal BCR (76.1%) was much higher than abnormal PTSSEPs (37.3%) and PDEPs (29.9%) among neurogenic ED which is expected, since the afferent and efferent nerve of BCR test are pudendal nerves.

Duplex ultrasonography is the most reliable and less invasive diagnostic modality for assessing ED. The most important parameters are the peak systolic velocity (PSV) and end-diastolic velocities (EDV) measured in the central penile arteries. PSV equal 35cm/sec or greater indicates normal arterial response to adequate pharmacological stimulation, whereas PSV below 25cm/sec indicate arterial insufficiency; intermediate values are not specific (30). The EDV and the corresponding semi-quantitative measurement of the RI may be informative about penile veno-occlusion. An EDV >5cm/sec combined with a normal arterial response is accepted as the measurement at which a venous leak is present (31). In our study, Bimix of 15mg papaverine and 1mg phentolamine was injected intracavernously to relax the vasculature as other reports (10), and the same evaluation criterion as above mentioned was applied; we found that ED caused by pelvic fracture had a vasculogenic etiology in 45.8% (55 of 120) of patients, of them 12.7% were subclassified as arteriogenic, 56.4% venogenic and 30.9% arteriovenogenic. Penile venous leakage occurred in 5 patients (Figure-3).

There are some limitations in our study. First, since invasive arteriography could not be accepted by most patients in our department, we could not determine whether the patients who showed a normal penile vascular response in ultrasound suffered extensive arterial lesions in the pudendal axis. Second, the three electrophysiological tests used in our study only accessed the large fiber functions (mainly A_β_or A_δ_); a careful study of neurogenic etiology in ED must necessarily assess the small fiber (c-fiber) pathways which might be detected with heat stimuli or capsaicin. These points might be further resolved in our future studies.

## CONCLUSIONS

ED is very common in patients with pelvic fractures associated with urethral injury. Neurovacular injuries contribute to the occurrence of ED. The neurogenic factor is the main etiology in relation to the vascular factor. Venous impotence is more common than arteriogenic ED. Most of the neurogenic ED patients had abnormal BCR outcomes. Our findings provide a detailed profile for the etiology of ED in patients after pelvic fracture.
